# miR-26a is Involved in Glycometabolism and Affects Boar Sperm Viability by Targeting *PDHX*

**DOI:** 10.3390/cells9010146

**Published:** 2020-01-08

**Authors:** Wencan Wang, Kai Liang, Yu Chang, Mingxia Ran, Yan Zhang, Malik Ahsan Ali, Dinghui Dai, Izhar Hyder Qazi, Ming Zhang, Guangbin Zhou, Jiandong Yang, Christiana Angel, Changjun Zeng

**Affiliations:** 1 College of Animal Sciences and Technology, and Farm Animal Genetic Resources Exploration and Innovation Key Laboratory of Sichuan Province, Sichuan Agricultural University, Chengdu 611130, China; wencan_wang1208@163.com (W.W.); sicau-liangkai@hotmail.com (K.L.); changy90s@163.com (Y.C.); 18227585649@163.com (M.R.); yanzhang@sicau.edu.cn (Y.Z.); Malik364ahsan@hotmail.com (M.A.A.); 71317@sicau.edu.cn (D.D.); vetdr_izhar@yahoo.com (I.H.Q.); zhm3000@126.com (M.Z.); zguangbin@sicau.edu.cn (G.Z.); yangjd@sicau.edu.cn (J.Y.); 2 Department of Theriogenology, Riphah College of Veterinary Sciences, Lahore 54000, Pakistan; 3 Department of Veterinary Anatomy & Histology, Shaheed Benazir Bhutto University of Veterinary and Animal Sciences, Sakrand 67210, Pakistan; 4 College of Veterinary Medicine, Sichuan Agricultural University, Chengdu 611130, China; qazi5502@yahoo.com; 5 Department of Veterinary Parasitology, Faculty of Veterinary Sciences, Shaheed Benazir Bhutto University of Veterinary and Animal Sciences, Sakrand 67210, Pakistan

**Keywords:** boar sperm, glycometabolism, miR-26a, *PDHX*, sperm viability

## Abstract

miR-26a is associated with sperm metabolism and can affect sperm motility and apoptosis. However, how miR-26a affects sperm motility remains largely unknown. Our previous study indicated that the *PDHX* gene is predicted to be a potential target of miR-26a, which is responsible for pyruvate oxidative decarboxylation which is considered as a key step for connecting glycolysis with oxidative phosphorylation. In this study, we first reported a potential relationship between miR-26a and *PDHX* and their expressions in fresh, frozen-thawed, and epididymal boar sperm. Then, sperm viability and survival were determined after transfection of miR-26a. mRNA and protein expression level of PDHX in the liquid-preserved boar sperm after transfection were also determined by RT-qPCR and Western Blot (WB). Our results showed that expression level of *PDHX* was significantly increased during sperm transit from epididymal caput to corpus and cauda. Similarly, expression of *PDHX* was significantly higher (*P* < 0.05) in fresh sperm as compared to epididymal cauda and frozen-thawed sperm. However, the expression of miR-26a in epididymal corpus sperm was significantly higher (*P* < 0.05) than that of caput and cauda sperm. Furthermore, after transfection of boar sperm with miR-26a mimic and inhibitor under liquid storage, the lowest and highest sperm viability was observed in miR-26a mimic and inhibitor treatment (*P* < 0.05), respectively. The protein levels of *PDHX*, after 24 and 48 h of transfection of miR-26a mimics and inhibitor, were notably decreased and increased (*P* < 0.05), respectively, as compared to negative control (NC) group. In conclusion, the novel and enticing findings of our study provide a reasonable evidence that miR-26a via *PDHX*, a link between glycolysis and oxidative phosphorylation, could regulate the glycometabolic pathway which eventually affect boar sperm viability and survival.

## 1. Introduction

Following spermatogenesis in testes, sperm undergo distinct post-gonadal phases of differentiation and maturation while transiting in the epididymis and subsequently attain maturity and progressive motility [1,2]. It has been suggested that under liquid storage conditions sperm exhibit morphological and functional changes similar to natural aging process along with apoptotic-like changes [3,4]. Cryopreservation is believed to result in structural and functional losses in sperm, leading to decreased overall sperm quality and fertilizing ability [5,6]. It has also been demonstrated that long-term cryopreservation of sperm significantly affects the motility-related parameters, apoptotic ratio and oxidation levels compared to those that are exposed to short-term cryopreservation [7]. Similarly, the metabolic activity of frozen-thawed sperm is inhibited, which contributes to the decreased sperm motility [8]. Sperm metabolism is an essential process by which sperm obtain energy via utilizing the substances dissolved in seminal plasma and cytosol. Furthermore, it plays an important role in maintaining normal function of sperm such as motility, survival and fertility [9,10]. Therefore, the metabolic activity of sperm is crucial for sustaining its survival and movement. However, due to the lower endogenous sugar content, sperm rely mainly on the exogenous sugar sources to fulfill energy requirements [11,12].

Sperm utilizes two metabolic pathways for producing energy, namely glycolysis and oxidative phosphorylation [13]. The sperm’s preference for energy metabolic pathways is believed to be species-specific. Sperm of different species prefer different sugar sources for glycolysis. For instance, pig and human sperm prefer glucose over fructose and dog’s sperm prefers fructose to produce pyruvate [12,14,15]. Under anaerobic conditions, the pyruvate can be converted to lactic acid via lactate dehydrogenase (LDH). However, under aerobic conditions, pyruvate oxidative decarboxylation catalyzed by pyruvate dehydrogenase complex (PDHC) which is a key step in connecting glycolysis with oxidative phosphorylation [16]. Specifically, pyruvate is transported into the mitochondrial matrix via voltage-gated channels and mitochondrial pyruvate carrier (MPC) complex in the outer and inner mitochondrial membrane respectively, where pyruvate undergoes oxidative decarboxylation in the presence of pyruvate dehydrogenase (PDH) to produce acetyl-CoA, one of the substrates of tricarboxylic acid cycle [17]. E3-binding protein (E3BP) is a structural subunit of PDHC, which is coded by pyruvate dehydrogenase complex component X (*PDHX*) [18,19]. E3BP shows a higher degree of homology to dihydrolipoamide transacetylase (E2), which plays a critical role in linking two catalytic subunits i.e., E2 and dihydrolipoamide dehydrogenase (E3) [20]. Although E3BP does not seems to have a catalytic function, it is essential for PDHC for performing its normal function [21]. Previous studies have shown that, patients with metabolic lactic acidosis, developmental retardation, and neurodegenerative diseases, exhibited defects and mutations in genes implicated in PDH pathway [22]. In 1990, Robinson and colleagues reported E3BP deficiency for the first time [23]. Since then, numerous studies have demonstrated that the occurrence of mutations in *PDHX* gene are due to PDH deficiency [24,25,26]. In addition, *PDHX* is inhibited by miR-27b and regulates the metabolic proliferation of breast cancer cells, which leads to reduced patient survival [27].

Species-specific differences seem to exist in energy metabolism, glycolysis and oxidative phosphorylation pathways in sperm. For instance, sperm motility and fertilizing ability of bulls and sheep decreases after inhibiting the mitochondrial respiratory chain, suggesting that the oxidative phosphorylation may serve as a major energy metabolism pathway in both species [28,29]. Guinea pigs have higher rates of aerobic respiration and glycolysis [30]. Similarly, human and mouse sperm rely mainly on glycolysis for producing ATP and resultant energy [31,32]. However, the energy metabolism pathway in boar sperm is still incompletely understood and remains as a matter of ongoing debate. Some researchers have argued that glycolysis is the main energy metabolism pathway for boar sperm [33]. However, Nevo and colleagues [34] have reported that, under anaerobic conditions, even in the presence of glucose and fructose, boar sperm showed no progressive motility, and only a slight flagellar swing was observed, indicating that glycolysis alone was not sufficient to fulfill the energy requirements, and therefore highlighting that oxidative phosphorylation might be an essential metabolic pathway adopted by boar sperm.

It is well known that microRNAs (miRNAs) act as the important post-transcriptional regulators by inhibiting the mRNA translation or by modulating the mRNA degradation, and only a few miRNAs have been found to be implicated in regulating the sperm motility, such as let-7a, -7d, -7e and miR-22 [35]. In addition, miRNAs can also affect boar sperm motility via regulating sperm apoptosis. Apoptosis plays a vital role in the process of differentiation of germ cells into mature sperm and eventually participate in fertilization and decay of these sperm [36,37]. Sperm apoptosis has an adverse effect on sperm vitality both in in vivo and in vitro and is considered as one of the critical factors affecting the fecundity in humans and animals [38,39,40]. It was reported that let-7g-5p can regulate the apoptosis in boar sperm by targeting *PMAIP1* gene, resulting in low motility of sperm [41]. miR-98, miR-181, miR-19, miR-504, and miR-676 are also involved in sperm apoptosis by regulating their target genes such as *FAS*, *Bcl-2*, *PTEN*, and *p53*, respectively, and thereby affecting the sperm motility and survival [42]. Furthermore, it has bene reported that down-regulated let-7b-5p can repress sperm glycolysis by targeting *AURKB* and results in decreased sperm motility [43].

miR-26a is a functional miRNA which is widely expressed in different bodily tissues [44,45,46], and also plays a vital role in regulating sperm metabolism and apoptosis. Huang et al. [47] reported that miR-26a affects the semen quality of Holstein bulls by negatively regulating the expression of phosphoenolpyruvate carboxykinase-1 (*PCK1*), a main gene involved in glycometabolism. Moreover, transcriptomic analysis has suggested that miR-26a is also implicated in bull sperm motility [48]. However, miR-26a regulates boar sperm apoptosis by targeting *PTEN* gene and has a link with decreased sperm motility [41]. Intriguingly, it has been reported that the expression level of miR-26a in highly motile frozen-thawed sperm was significantly higher as compared to the low-motile frozen-thawed sperm [49]. These results provide reasonable evidence that miR-26a may be involved in regulating the sperm metabolism and apoptosis, and in turn can affect sperm motility and survival. However, whether there are other metabolic regulatory pathways of miR-26a exist or not requires further exploration.

In our previous work, we have shown that miR-26a is related to boar sperm motility [42]. However, whether *PDHX* is involved or not, as a bridge between glycolysis and oxidative phosphorylation, in the regulation of sperm energy metabolism, motility and survival remains largely unknown. Thus, we have first determined the link between miR-26a and *PDHX* gene by dual luciferase assay, and then expressions of *PDHX* and miR-26a were evaluated in boar epididymal (caput, corpus, and cauda), fresh, and frozen-thawed sperm. Finally, we transfected miR-26a into boar sperm, and then viability, mRNA and protein expression of *PDHX* of sperm were evaluated under liquid storage conditions.

## 2. Materials and Methods

### 2.1. Animal Ethics Statement

Seven healthy and sexually mature Landrace boars were humanely euthanized by electrical stunning method to minimize all means of discomfort. All procedures were carried out in strict accordance with the Regulations of the Administration of Affairs Concerning Experimental Animals (Ministry of Science and Technology, China, revised in June 2004) and approved by the Institutional Animal Care and Use Committee in the College of Animal Science and Technology, Sichuan Agricultural University, Sichuan, China, under permit No. DKY2018202013.

### 2.2. Sperm Collection and Treatment

Immediately after euthanasia of each boar (*n* = 7), epididymides were carefully collected and processed within three hours. Boar sperm from epididymal caput, corpus, and cauda were collected according to the procedures described by Saenz et al. [50] with some modifications. Briefly, sperm were flushed from epididymis using RNase-free PBS and were immediately frozen in liquid nitrogen and stored at −80 °C. Fresh sperm samples (*n* = 5) were collected by a manual collection method from five sexually mature Landrace boars. Only ejaculates exhibiting more than 80% sperm motility, normal morphology, and above 1 × 10^8^ mL^−1^ concentration of sperm were used. Then, these ejaculates were equally divided into three treatment aliquots. The first aliquot was directly frozen in liquid nitrogen and then stored at −80 °C; second aliquot was directly diluted for transfection experiment, and third aliquot was cryopreserved.

### 2.3. Primers Design

All primers were designed according to counterparts in GenBank using NCBI Primer-Blast search or from published literatures (Table 1). *U6* and *GAPDH* were used as reference genes for miRNA and mRNA expression analyses, respectively.

### 2.4. Target Prediction of miR-26a and Dual Luciferase Reporter Assay

Four tools, TargetScan [51], miRanda [52], miRWalk [53], and picTar [54] were used to predict the target genes of miR-26a based on the seed region of miR-26a and 3′-UTR of *PDHX*. The wild type vector (pWT-*PDHX*) is the 3′-UTR region of *PDHX* gene including miR-26a binding site. The mutant vector (pMT-*PDHX*) was obtained by mutating a few bases of the binding site.

Hela cells were cultured in Dulbecco’s modified Eagle’s medium (DMEM) supplemented with 10% fetal bovine serum (FBS) at 37 °C in a humidified atmosphere containing 5% CO_2_. Each well was seeded with 1 × 10^4^ cells in 96-well plates. When cell confluence reached 70%, the pWT-*PDHX* and pMT-*PDHX*, were co-transfected with miR-26a mimic and mimic NC (RiboBio, Guangzhou, China), respectively, by using FuGENE HD Transfection Reagent (Promega, Madison, WI, USA) following the manufacturer’s protocol. After 48 h, cell lysates were prepared and relative dual-luciferase activity was detected by a Dual-Luciferase Reporter Assay System (Promega, Madison, WI, USA).

### 2.5. Sperm Liquid Storage, Cryopreservation, and Electrotransfection

Initially, an isothermal and equal-volume Beltsville thawing solution (BTS: 3.7 g glucose, 0.3 g trisodium citrate, 0.125 g Na_2_-EDTA, 0.125 g NaHCO_3_, 0.075 g KCl, 0.6 g/L penicillin G sodium, and 1.0 g/L dihydrostreptomycin; all diluted to 100 mL) was added to the fresh boar semen. Following gentle mixing, it was wrapped in a towel, kept at room temperature for 30 min, and finally stored in a refrigerator at 17 °C. Ejaculated boar sperm were cryopreserved according to our laboratory’s protocol [55]. Briefly, sperm were diluted (1:1 *v*/*v*) with BTS and cooled slowly to 17 °C. The diluted sperm were centrifuged at 239 *g* for 6 min. Then, the supernatant was discarded, and sperm pellets were suspended in lactose-egg yolk extender (LEY: 11g β-lactose and 20 mL hen’s egg yolk were added to 80 mL ddH_2_O), and cooled down slowly to 4 °C in 4 h. Subsequently, the same volume of second extender (lactose-egg yolk supplemented with glycerol and the concentration of glycerol (6%) was added to above mixture for getting a final concentration of 3% glycerol. The completely miscible liquids were packed into 0.25 mL straws (FHK, Tokyo, Japan) and equilibrated at approximately −150 °C above liquid nitrogen (LN) vapor for 15 min, then the straws were placed into LN (−196 °C) for long-term storage.

Electrotransfection was performed according to Zhang’s electrotransfection method [56]. Ejaculated boar sperm were diluted to a concentration of 5 × 10^7^ mL^−1^ using BTS. Transfection was conducted using a Cell Manipulation ECM-2001 (BTX, Holliston, MA, USA) with 25 nM of miRNA mimic and inhibitor or a scrambled negative control (NC) (RiboBio, Guangzhou, China) according to the manufacturer’s instructions and pulse conditions were adjusted as 4 × 300 V for 100 μs. The sperm were cultured under the same condition after transfection. The efficiency of transfection was detected at 6 h, and then sperm viability, relative expression of miR-26a, and *PDHX* were assessed every 24 h until 96 h. WB was performed at 24 h and 48 h after transfection.

### 2.6. Sperm Viability Detection

Sperm viability was determined by trypan blue staining after electro-transfection. Briefly, 20 µL semen and an equal volume of 0.8% trypan blue were uniformly mixed. After incubation for 5 min at 37 °C, at least 200 live sperms were counted according to manufacturer’s instruction (live sperms were unstained and dead ones were stained). All procedures were repeated for three times.

### 2.7. Total RNA Extraction, cDNA Synthesis and RT-qPCR

Total RNA from boar sperm was extracted with Trizol LS Reagent (Invitrogen, Carlsbad, CA, USA) according to our laboratory’s protocol. The somatic cells in all sperm samples were removed according to the procedures described by Bianchi et al. [57] with some modifications. Briefly, boar sperms were incubated with 0.1% SDS and 0.5% Triton X-100 (Coolaber, Beijing, China) on ice for 15 min. The concentration and quality of total RNA were measured with a Nanodrop 2000 (Thermo Fisher Scientific, Wilmington, DE, USA). Reverse transcription of total RNA and miRNA was performed by using TaKaRa PrimeScript RT Reagent Kit (Takara Biotech, Dalian, China) and Takara SYBR PrimeScript miRNA RT-PCR Reagent Kit (Takara Biotech, Dalian, China), respectively according to the manufacturer’s instructions. All reverse transcription products were preserved at −20 °C.

RT-qPCR was performed using SYBR Premix Ex Taq II Reagent Kit (Takara Biotech, China) on the CFX 96 Real-Time PCR Detection System (Bio-Rad, Hercules, CA, USA). Briefly, RT-qPCR was carried out using 5 µL SYBR Green I Premix, 0.5 µL each of forward and reverse primers, and 1 µL of cDNA, with the addition of RNase-free water to make a total volume of 10 µL. Thermal cycling was performed as follows: an initial step of denaturation at 95 °C for 3 min, 40 cycles of amplification at 95 °C for 5 s, and the primer specific annealing temperatures were implied for 30 s. Relative expression levels were determined using 2^−∆∆CT^ method [58].

### 2.8. Western Blot Analysis

Western blot (WB) analyses was performed as described previously [59] with some modifications. Briefly, total protein was extracted from sperm in sodium dodecyl sulfate (SDS) sample buffer. The protein was separated by 12% SDS-PAGE and transferred to PVDF membrane (Roche, Basel, Switzerland). Non-specific binding sites of protein were blocked by incubation in Tris-Buffered Saline Tween-20 (TBST) containing 0.1% (*v*/*v*) Tween-20 and 5% (*w*/*v*) Bovine Serum Albumin (BSA) (Thermo Fisher Scientific, Wilmington, DE, USA). The membranes were immunoblotted with anti-PDHX and anti-β-tubulin (ZEN BIO, Chengdu, China) and diluted in TBST with 5% BSA (β-tubulin 1:10,000 and PDHX 1:500 dilution) overnight at 4 °C. Followed by incubation with goat anti-rabbit IgG (ZEN BIO, Chengdu, China) for PDHX and β-tubulin, 1:10,000 dilution. After washing in TBST, enhanced chemiluminescence (ECL) detection was performed by using Immun-Star™ WesternC™ Chemiluminescence Kit (BIO-RAD, Hercules, CA, USA) according to the manufacturer’s specifications. The exposure and development of PVDF membrane were performed using ChemiScope 6000 Exp (CLiNX, Shanghai, China), and band intensities were analyzed using a Gel-Pro Analyzer (Media Cybernetics, Bethesda, MD, USA).

### 2.9. Statistical Analysis

All data were tested for normality and variance homogeneity prior to statistical analysis. The relative gene expressions and protein levels were analyzed by Student *t*-test and sperm viability was analyzed by ANOVA followed by a Tukey post-hoc test. Differences were considered significant when *P <* 0.05 and the results are shown as mean ± SEM.

## 3. Results

### 3.1. PDHX is Direct Target Gene of miR-26a

**Figure 1:**
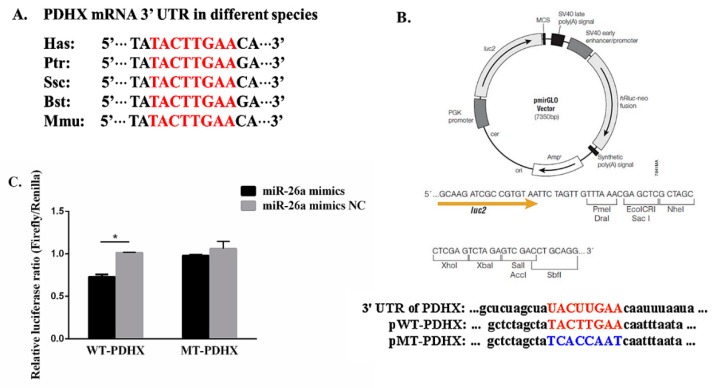
Target gene prediction of miR-26a and dual-luciferase reporter assay. (**A**) The predicted binding sites between pyruvate dehydrogenase complex component X (*PDHX*) 3’-UTR and miR-26a in various species. (**B**) The structure of recombinant plasmid and the binding site of wild type (WT) (red colored) and mutant type (MT) (blue colored) between *PDHX* 3’-UTR and miR-26a. Firefly luciferase and Renilla luciferase were regarded as the report gene and reference gene, respectively. (**C**) The result of dual-luciferase reporter assay. “*” indicates statistical significance at *P* < 0.05.

The prediction results showed that *PDHX* 3′-UTR harbors only one potential target site for miRNA-26a based on TargetScan, miRWalk, miRanda, and picTar. The sequence of the target site was highly conserved in *Homo sapiens* (Hsa), *Pan troglodytes* (Ptr), *Sus scrofa* (Ssc), *Bos taurus* (Bst), and *Mus musculus* (Mmu) (Figure 1A)

The dual luciferase recombinant, wild type and mutant type plasmid sequences of *PDHX* 3’-UTR are shown in Figure 1B. Recombinant plasmid with WT and MT *PDHX* 3’-UTR were co-transfected with miR-26a mimic and mimic NC, respectively into Hela cells. Based on dual-luciferase reporter assay, miR-26a significantly decreased (*P* < 0.05) the relative luciferase ratio in pWT group as compared to the mimic NC group, while no noticeable difference (*P* > 0.05) was found in pMT group (Figure 1C).

**Figure 2:**
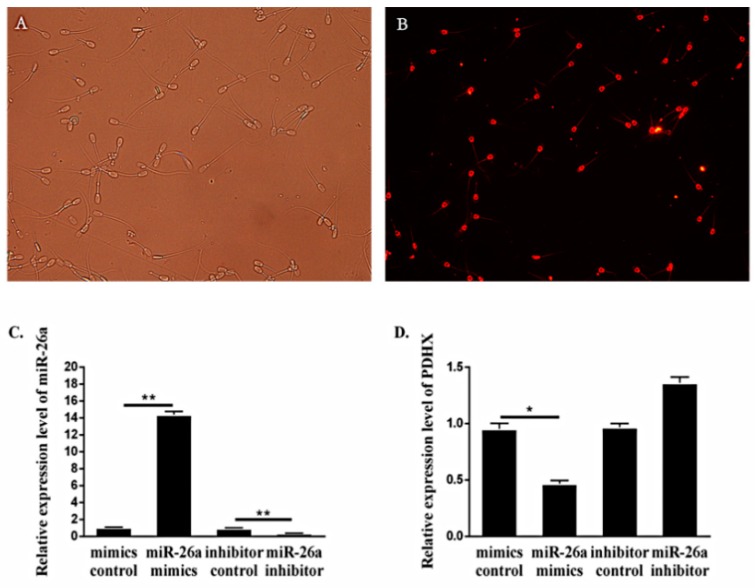
The transfection efficiency and expression level of miR-26a and *PDHX*. (**A**,**B**) Sperm transfected with mimic negative control (NC) with Cy3 fluorophore under white light conditions and fluorescence conditions, 200×. (**C**,**D**) The expression of miR-26a and *PDHX* after transfection of 6 h, respectively. “*” indicates statistical significance at *P* < 0.05 and “**” indicates statistical significance at *P* < 0.01.

To further determine the transfection efficiency and relationship between *PDHX* and miR-26a, mimic NC with Cy-3 fluorophore was transfected into boar sperm for 6 h. The transfection efficiency was about 70%, which was calculated by counting the proportion of sperm with red fluorescence signals (Figure 2A,B). The expression of miR-26a in boar sperm transfected with miR-26a mimic was significantly elevated (*P* < 0.01) as compared to mimic NC group, and the expression in inhibitor group was significantly lower (*P* < 0.01) than the inhibitor NC group (Figure 2C). Besides this, the expression of *PDHX* was decreased when miR-26a was overexpressed (Figure 2D). Therefore, these findings provide reasonable evidence that *PDHX* is a direct target gene of miR-26a and indicate that miR-26a may have potential implication in inhibiting the expression of *PDHX* at the mRNA level.

### 3.2. Expression of miR-26a and PDHX in Epididymal Sperm

**Figure 3:**
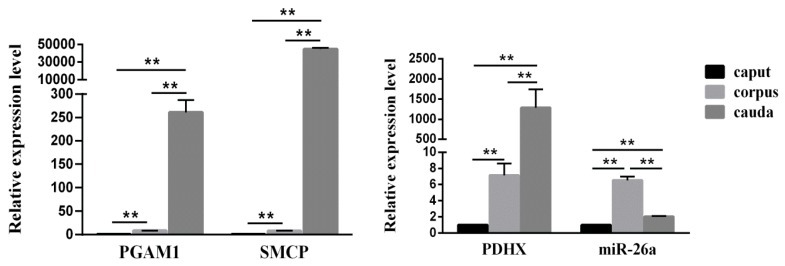
The expression levels of *PGAM1*, *SMCP* (left) and miR-26a, *PDHX* (right) in the epididymal caput, corpus and cauda sperm. “**” indicates statistical significance at *P* < 0.01.

We first detected the expression of two energy metabolism-related genes i.e., *PGAM1* and *SMCP* in boar epididymal sperm. The RT-qPCR results showed a significant difference (*P* < 0.01) in the expression level of *PGAM1* and *SMCP* in sperm retrieved from different epididymal segments (caput, corpus and cauda). Next, we determined the relative expression of *PDHX* in epididymal caput, corpus and cauda sperm which showed similar results to that of *PGAM1* and *SMCP* (Figure 3). However, the expression level of miR-26a in the epididymal caput, corpus and cauda did not correspond exactly to the trend shown in the expression of *PDHX*. Moreover, miR-26a showed the highest expression in epididymal corpus sperm and it was significantly (*P* < 0.05) higher than the caput and cauda sperm, indicating that sperm motility might gradually increase when sperm transit in epididymis from the caput to cauda.

### 3.3. Comparison of miR-26a and PDHX Expression in Epididymal cauda, Ejaculated and Frozen-Thawed Boar Sperm

**Figure 4:**
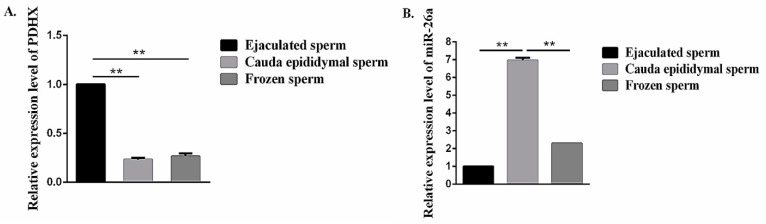
The expression level of miR-26a and *PDHX* in the epididymal cauda, ejaculated sperm and frozen-thawed sperm. (**A**) *PDHX*. (**B**) miR-26a. “**” indicates statistical significance at *P* < 0.01.

Our RT-qPCR analysis showed that *PDHX* was abundantly (*P* < 0.01) expressed in ejaculated sperm, and no significant difference (*P* > 0.05) in expression was observed between epididymal cauda and frozen-thawed sperm (Figure 4A). Moreover, the expression level of miR-26a in epididymal cauda sperm was significantly higher (*P* < 0.01) as compared to ejaculated and frozen-thawed sperm (Figure 4B).

### 3.4. Effects of miR-26a Transfection on PDHX Expression and Boar Sperm Viability Under Liquid Preservation Conditions

**Figure 5:**
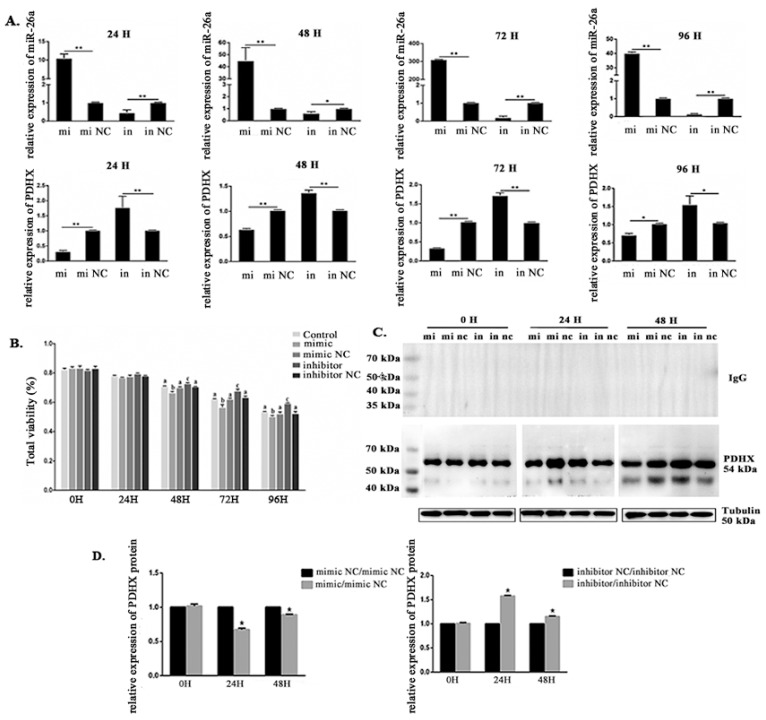
The expression level of miR-26a and *PDHX*, and sperm viability in miR-26a transfected boar sperm at 17 °C. (**A**) Sperm viability after miR-26a transfection. (**B**) The expression level of miR-26a and *PDHX* after transfection with miR-26a mimic, mimic control, miR-26a inhibitor and inhibitor control for 24 h, 48 h, 72 h and 96 h, respectively. (**C**) WB analysis after 0 h, 24 h, and 48 h treatment with miR-26a mimic/mimic NC and inhibitor/inhibitor NC showing the expression of PDHX and β-tubulin. The negative control rabbit IgG in place of the primary antibody was used. (**D**) The relative protein level of PDHX after transfection with miR-26a mimic and inhibitor. Values were normalized using β-tubulin protein as an internal reference, and then mimic NC and inhibitor NC groups were used as references to measure the relative expression levels. mi: mimic; mi nc: mimic NC; in: inhibitor; in nc: inhibitor NC. “*” indicates statistical significance at *P* < 0.05 and “**” indicates statistical significance at *P* < 0.01. Different alphabets indicate statistical significance at *P* < 0.05.

During liquid preservation of boar sperm, we have observed that the expression level of miR-26a was first increased and then decreased. Intriguingly, the expression level of miR-26a was significantly increased when sperm were transfected with miR-26a mimic and decreased following miR-26a inhibitor transfection. Conversely, the expression of *PDHX* with miR-26a mimic treatment was significantly decreased; however, it increased following miR-26a inhibitor transfection (Figure 5A).

Sperm viability was evaluated at 0 h, 24 h, 48 h, 72 h, and 96 h by using 0.4% Trypan Blue staining as described earlier. The results showed that sperm viability has gradually decreased with increasing storage time. Of note, sperm viability was around 80% at 24 h, and each treatment showed no significant difference when compared to the control group. However, 48 h later, sperm viability in miR-26a mimic transfected treatments were significantly lower (*P* < 0.05) compared to the control group. Conversely, sperm viability in miR-26a inhibitor transfected treatments were significantly higher (*P* < 0.05) as compared to control group. Moreover, sperm viability was decreased to about 60% at 96 h (Figure 5B).

Further, WB analysis indicated that the protein level of PDHX was significantly (*P* < 0.05) decreased and increased after 24 h and 48 h transfection with miR-26a mimics and inhibitor, respectively, as compared to NC group (Figure 5C,D).

## 4. Discussion

Although previously it has been shown that miR-26a could affect glucose metabolism in cancer cells by targeted-regulation of expression of *PDHX* mRNA and protein [60]. So far, there was no report on the regulatory relationship between miR-26a and *PDHX* which can affect sperm glycometabolism and thus the survival of boar sperm. Our current study provides a novel evidence that miR-26a is directly linked with *PDHX* in boar sperm and might regulate the energy metabolism and affect sperm viability. We observed that the expressions of miR-26a and *PDHX* exhibit significant differences among epididymal (caput, corpus and cauda), ejaculated and frozen-thawed sperm. Moreover, previous studies have also demonstrated the differential metabolism and viability percentage in epididymal, ejaculated and frozen-thawed sperm [61,62]. Following transfection with miR-26a mimic, the mRNA expression level of *PDHX* was significantly down-regulated, and a decreased sperm viability was also observed in our study. Meanwhile, the protein levels of *PDHX* transfected with miR-26a mimic and inhibitor after 24 h and 48 h were also significantly changed. It was reported that, under the condition of low current, electrotransfection has no significant impact on viability, plasma membrane, acrosomal and DNA integrity of boar sperm [63]. Furthermore, the ram sperm viability remained about 80% after electrotransfection [56]. In present study, no significant variation of mRNA and protein level of PDHX were found before and after electrotransfection without miR-26a mimic and inhibitor. Reversely, when sperms were electrotransfected with miR-26a mimic and inhibitor and evaluated after 24 h and 48 h, significant changes of mRNA and protein levels of *PDHX* were observed. This suggested that electrotransfection was an effective method and have no impact on the expression of *PDHX*. Our findings provide a reasonable molecular evidence and reveal a potential link between miR-26a/*PDHX* and sperm viability.

*PGAM1* (phosphoglycerate mutase 1) and *SMCP* (sperm mitochondria associated cysteine rich protein) are two important energy metabolism-related proteins of sperm. *PGAM1* catalyzes the conversion between 3-phosphoglyceric acid and 2-phosphoglyceric acid which regulates the glycolytic pathway and is also involved in multiple cancer processes [64,65]. Moreover, *SMCP* is a specific protein of male germ cells, located in the outer membrane of sperm mitochondria [66]. Knockout of *SMCP* could lead to decreased sperm motility and male infertility [67]. In present study, the expression levels of sperm *SMCP* and *PGAM1* were gradually increased during sperm transition in the epididymal caput, corpus and cauda. Similar results were found for *PDHX* expression in the epididymal caput, corpus and cauda sperm. Thus, our results are coherent with the fact that sperm motility and metabolism gradually increase during sperm transit in epididymis. However, it is still incompletely understood that which metabolic pathway is used by boar sperm for producing ATP which is required for maintaining its movement and longevity. It is generally believed that balance of sperm metabolic pathways is controlled by external factors such as environmental substrate composition and oxygen levels, suggesting that metabolic events in sperm may be affected by changes in environmental conditions [68]. It has been suggested that both glycolysis and oxidative phosphorylation are necessary for maintenance of normal sperm function and fertilizing ability; however, these two pathways can act in tandem as well [69]. In some animal species, mitochondrial oxidative phosphorylation is considered as an essential pathway for sperm energy metabolism [28,29]. Similarly, in our study, we also envisaged that increased expression of *PDHX* may be linked with corresponding increase in the mitochondrial oxidative phosphorylation level in boar sperm.

Surprisingly, the expression levels of miR-26a in epididymal caput, corpus and cauda sperm were not well consistent with the increasing expression level of its target gene i.e., *PDHX*. Therefore, keeping these differences in mind, we envisage that there may be other putative regulatory factors which might be implicated in modulating miR-26a/*PDHX* interaction. Recent studies have reported that epididymal exosomes, also called epididymosomes, a kind of vesicles contained complex protein component and RNAs which are secreted by epididymal epithelial cells and could transfer RNAs, miRNAs, small molecules and exosome-associated proteins through the plasma membrane of immature sperm, and are involved in regulating sperm motility and maturation process [70,71,72,73,74,75,76,77]. In mouse model, it has been demonstrated that expression profile of sperm RNAs could change during maturation of epididymal sperm and this change was caused by epididymosomes [78,79]. The similar exosomes have also been identified in other species such as amphibians [80]. However, protein composition of epididymosomes could vary in epididymal caput, corpus and cauda. Proteomic analysis of the epididymosomes has revealed that most of these proteins could play a crucial role in transfer and metabolism of exosome component. In addition, some new proteins of epididymosomes which are associated with sperm fertilization have also been identified [81]. Moreover, exosomes are also present in seminal plasma which interact with sperm and have been implicated in regulating the changes in miRNAs [82]. It has been demonstrated that there are some prostasomes (a kind of exosome in seminal plasma) which can specifically deliver the genetic and bioactive materials to sperm, and subsequently can improve the quality of semen and the rate of fertilization in boar [83,84,85]. It is possible that expression pattern of miR-26a and *PDHX* might be effected by epididymosomes and confounding bio-factors during transit of sperm in epididymis. Therefore, further functional focused and mechanistic studies have the real potential to elucidate these mechanisms and improve our understanding in this regard.

In addition to exosomes, methylation level of miRNA itself or its promoter region is also important factor which is implicated in affecting miRNA expression. It was also reported that CpG Island of miRNA gene promoter exhibits a methylation modification, resultantly blocking the transcription of miRNA [86]. Moreover, following downregulation of expression of an RNA demethylase FTO, the expression levels of several miRNAs were affected. These observations were further confirmed by the presence of a N^6^-methyladenosine (m^6^A), a type of RNA methylation in miRNA [87]. Besides these, other similar studies have also demonstrated that the regulation of expression of 5-methylcytosine (m^5^C) or m^6^A methylation-related genes could affect processing of pre-miRNAs [88,89]. The presence of m^6^A modification in pri-miRNAs may be an important signaling marker for the processing of pri-miRNAs into the mature miRNAs, which is involved in the regulation of miRNAs biogenesis [90]. Although specific DNA promoter sequences of sperm are methylated in different segments of epididymis of mouse (initial segment, caput, corpus and cauda), however, these promoter methylation modifications were found to have no significant effect on gene transcription [91]. Nevertheless, questions such as: whether regionally specific methylation modifications exist in the boar epididymis or not, and these modifications further interact with exosomes or not, to influence the methylated modification pattern of genome or RNAs of boar sperm during sperm transit in the epididymis. Another ambiguity is that whether these modifications are implicated or not in affecting the expression level of miR-26a and *PDHX*. To answer these questions further in-depth functional and mechanistic studies are required.

## 5. Conclusions

Our study is the first to report that miR-26a by targeting *PDHX*, a link between glycolysis and oxidative phosphorylation, might regulate the glycometabolic pathway and eventually affect the boar sperm viability and survival. Additionally, our results also provide reasonable evidence that mitochondrial oxidative phosphorylation might be an essential and indispensable metabolic pathway for maintenance of boar sperm motility and survival. However, further functional and mechanistic studies are still required for in-depth understanding of such molecular pathways which are implicated in energy metabolism in boar sperm.

## Figures and Tables

**Table 1:** Primers used for quantitative reverse transcription PCR (RT-qPCR).

Gene	Primer Sequence (5′→3′)	Tm (°C)	Size (bp)	GenBank Accession
*GAPDH*	F: ACTCACTCTTCTACCTTTGATGCT	60	100	AF017079
	R: TGTTGCTGTAGCCAAATTCA			
*PDHX*	F: TCACAGCACGCAGTCTCTTT	60	132	XM_003122869.3
	R: AAGGCATCTCCAGCACTCAC			
*SMCP*	F: CTGAGTGCACCTGCCTGAATA	60	106	AY788095.1
	R: TTGGACCCCTGTCTTGGACT			
*PGAM1*	F: GCGAGAGCCTGAAGGACACTATTG	60	138	XM_003483535.4
	R: CCTCCAGATGCTTGACGATGCC			
